# Biogels in Soils: Plant Mucilage as a Biofilm Matrix That Shapes the Rhizosphere Microbial Habitat

**DOI:** 10.3389/fpls.2021.798992

**Published:** 2022-01-13

**Authors:** Meisam Nazari, Samuel Bickel, Pascal Benard, Kyle Mason-Jones, Andrea Carminati, Michaela A. Dippold

**Affiliations:** ^1^Division of Biogeochemistry of Agroecosystems, Georg-August University of Göttingen, Göttingen, Germany; ^2^Physics of Soils and Terrestrial Ecosystems, Department of Environmental Systems Science, Institute of Terrestrial Ecosystems, ETH Zürich, Zurich, Switzerland; ^3^Department of Terrestrial Ecology, Netherlands Institute of Ecology (NIOO-KNAW), Wageningen, Netherlands; ^4^Geo-Biosphere Interactions, University of Tübingen, Tübingen, Germany

**Keywords:** biofilm, EPS, microorganism, mucilage, rhizosphere, root

## Abstract

Mucilage is a gelatinous high-molecular-weight substance produced by almost all plants, serving numerous functions for plant and soil. To date, research has mainly focused on hydraulic and physical functions of mucilage in the rhizosphere. Studies on the relevance of mucilage as a microbial habitat are scarce. Extracellular polymeric substances (EPS) are similarly gelatinous high-molecular-weight substances produced by microorganisms. EPS support the establishment of microbial assemblages in soils, mainly through providing a moist environment, a protective barrier, and serving as carbon and nutrient sources. We propose that mucilage shares physical and chemical properties with EPS, functioning similarly as a biofilm matrix covering a large extent of the rhizosphere. Our analyses found no evidence of consistent differences in viscosity and surface tension between EPS and mucilage, these being important physical properties. With regard to chemical composition, polysaccharide, protein, neutral monosaccharide, and uronic acid composition also showed no consistent differences between these biogels. Our analyses and literature review suggest that all major functions known for EPS and required for biofilm formation are also provided by mucilage, offering a protected habitat optimized for nutrient mobilization. Mucilage enables high rhizo-microbial abundance and activity by functioning as carbon and nutrient source. We suggest that the role of mucilage as a biofilm matrix has been underestimated, and should be considered in conceptual models of the rhizosphere.

## Introduction

Plant roots are the major organs responsible for water and nutrient uptake from soil. Methodological difficulties in sampling belowground traits result in a much more detailed understanding of above- than belowground plant ecophysiology (Oburger and Schmidt, 2016; McCormack et al., 2017). Roots exude a diverse set of compounds into the rhizosphere, including sugars, amino acids, and secondary metabolites, which regulate rhizosphere functions (Walker et al., 2003; Dutta et al., 2013). Mucilage is a gelatinous high-molecular-weight substance produced by almost all plants, comprising approximately half of root exudates (Chaboud, 1983). The mucilage backbone is built of polysaccharides, but proteins, minerals, and lipids are also part of the biogel (Nazari, 2021). So far, mucilage has mainly been recognized to have hydraulic, mechanical, and physical functions in the rhizosphere. For instance, mucilage increases the rhizosphere water content, improves plant water uptake under drought, reduces friction against the growing root, and stabilizes soil aggregates (Young, 1995; Czarnes et al., 2000; Iijima et al., 2003; Carminati et al., 2010; Ahmed et al., 2015). However, only a few studies have investigated the relevance of mucilage for microbial processes. For example, it has been indicated that microorganisms utilize mucilage as an energy source and habitat (Mary et al., 1993; Ahmed et al., 2018a,b). It has been reported that maize (*Zea mays* L.) crown root mucilage harbors nitrogen-fixing bacteria, which contribute to the fixation of a considerable amount of the plant’s nitrogen requirement (Van Deynze et al., 2018; Amicucci et al., 2019). Mucilage likely plays a central role in mediating plant-microbe interactions in the rhizosphere, but the magnitude of its relevance remains unclear.

Microorganisms can live planktonically, in suspended aggregates, and in attached biofilms (Flemming and Wuertz, 2019). Extracellular polymeric substances (EPS) produced by microorganisms are a three-dimensional matrix accounting for more than 90% of the dry mass of microbial biofilms (Flemming and Wingender, 2010). EPS are mainly composed of polysaccharides, but also contain proteins, nucleic acids, lipids, and minerals (Flemming and Wingender, 2010). EPS are formed upon the attachment of microorganisms to surfaces in order to establish biofilms (Fong and Yildiz, 2015; Jamal et al., 2018). It has been shown that EPS enhance the liquid phase viscosity compared to water and create an interconnected network (Stoodley et al., 2002; Flemming and Wingender, 2010; Volk et al., 2016). EPS improve soil water retention and liquid-phase connectivity (Rosenzweig et al., 2012; Benard et al., 2019), due to uronic acid-Ca^2+^ binding in their chemical structure (Aravamudhan et al., 2014). Alginate is an anionic polysaccharide found in EPS, consisting of only uronic acids such as glucuronic acid, galacturonic acid, and mannuronic acid (Sutherland, 2001; Van Hullebusch et al., 2004; Flemming and Wingender, 2010). Alginate participates in the formation of microcolonies at the beginning of the biofilm formation process, increases EPS hydration, and assists in trapping cations such as Ca^2+^, Zn^2+^, Cd^2+^, and Ni^2+^ (Wuertz et al., 2001; Van Hullebusch et al., 2004; Flemming and Wingender, 2010).

EPS constitute an important part of the carbon pool in soils that plays key roles in soil microbial ecology (Flemming and Wingender, 2010). However, a main function of EPS is to protect microorganisms against environmental stresses such as drought, acidity, or salinity (Kumar et al., 2007; Vardharajula and Sk, 2014). EPS improve soil moisture status in microbial hotspots like the rhizosphere (Kuzyakov and Blagodatskaya, 2015). EPS are capable of absorbing 15–20 times more water than their dry weight and thus strongly increase the water holding capacity of soils (Chenu, 1993; Adessi et al., 2018). EPS strongly influence interactions between bacteria and their viruses (bacteriophages) by binding virus particles and slowing down their movement (Vidakovic et al., 2018). EPS also facilitate chemical communications between microorganisms within the biofilm, leading to increased microbial turnover and element cycling (Joubert et al., 2006; Flemming et al., 2007). Furthermore, EPS in soil can enhance the exchange of genetic material between microorganisms, trap nutrients, protect microorganisms against antimicrobial factors, and act as a carbon source for microorganisms, but they are also a key component involved in soil aggregate formation and thus in the formation of further soil micro-habitats (Costa et al., 2018).

Plants may produce mucilage not merely for improving hydraulic, mechanical, and physical functions in the rhizosphere, but potentially also to function as a biofilm matrix and support a rapid establishment of dense symbiont microbial communities and high microbial activity in the rhizosphere. Mucilage has a high potential to function as a biofilm matrix by providing a moist environment, protective barrier, and carbon and nutrient source for microbial communities. This study analyzes and reviews existing evidence to determine whether plant mucilage and microbial EPS have comparable physical and chemical properties, leading to analogous biophysical and biochemical features. This indicates similar microbial habitat properties of both biogels. To support this perspective, viscosity and surface tension as important physical properties and total polysaccharide, total protein, neutral monosaccharide, and uronic acid proportions as important chemical properties of mucilage and EPS are compared. Furthermore, to assess its quantitative relevance, we estimate the extent of the “mucilage biofilm” along the root axis, including bioenergetic viewpoints of microbial advantages living in a “plant-provided” biofilm and discuss the implications of this biofilm matrix as a key prerequisite for the high microbial activity in the rhizosphere.

## Methodology

### Data Collection and Standardization

In total, 376 datasets were collected from 83 related papers published between 1974 and 2019. The online tool WebPlotDigitizer was used to extract data from the charts^1^. The viscosity and surface tension data were considered physical indices for the comparison of mucilage and EPS. To evaluate chemical properties of mucilage and EPS, total polysaccharide, total protein, neutral monosaccharide, and uronic acid proportions were compared. The investigated neutral monosaccharides included galactose, fucose, glucose, mannose, arabinose, rhamnose, and xylose, and uronic acids included glucuronic and galacturonic acid.

We considered some criteria for selection of the data. For the physical properties, the viscosity values had been measured at a solute concentration of 0.5 mg ml^–1^, shear rate of 0.5 s^–1^, and temperature of 20–25°C, and the surface tension values had been measured at a solute concentration of 0.5 mg ml^–1^ and temperature of 20–25°C. Only root and seed mucilage, which has rhizospheric relevance, were considered. All data related to the chemical properties were derived from pure mucilage and EPS. Data not fulfilling these criteria were excluded. SI units were used to standardize the viscosity (Pa s) and surface tension (N m^–1^) values. A list of plant and microbial species that produced the biogel, accompanied by their references, has been provided in Supplementary Material 1.

### Calculations of Spatial and Temporal Distribution of Mucilage and Bacterial Cell Abundances Around Growing Roots

To calculate the mucilage spatial and temporal distribution at a given exudation rate around a growing model root, we assumed a root-soil system with the following parameters: Soil porosity = 50%; crown root diameter = 3.3 mm (unpublished data of maize); mucilage exudation rate = 1.41 mg dry weight per day and root tip (unpublished data of the same maize plants); maximum hydration ratio of mucilage = 425:1 (wet mass: dry mass, unpublished data of the same maize plants), assuming a 39% water saturation upon exudation at the root tip and a rapid saturation to 100% within 6 h (Sealey et al., 1995); root elongation rate = 30 mm per day (Schmidt et al., 2013); and a maximum decomposition rate of the mucilage = 50% in 7 days (Ahmed et al., 2018a). Note that the diffusion of mucilage was neglected. Detailed equations and explanations of the mucilage spatial and temporal distribution model have been provided in Supplementary Material 2.

Considering bacteria as main mucilage consumers (Ahmed et al., 2018b), bacterial abundance in the rhizosphere was estimated. The mucilage available to bacterial degradation (*C*_*in*_) was assumed to produce bacterial biomass under carbon limitation. The growth and biomass yield of several rhizosphere bacteria using glucose are comparable to the growth achieved by mucilage as a sole carbon source (Knee et al., 2001). Here, we considered the carbohydrate fraction of mucilage obtained in this study (*f*_*c*_ = 0.77) to be available for bacterial consumption (considering the mass-fraction of carbon for simple sugars; e.g., glucose *w*_*c*_ = 0.4). The upper bound on mucilage-derived carbon (*C*_*in*_) that can be allocated to produce bacterial cell biomass (*M*_*B*_) was obtained by considering an average carbon use efficiency (CUE) for a range of carbon sources (*M*_*B*_ = *f_*c*_* × *w_*c*_* × CUE × *C*_*in*_). We assumed that maintenance costs were negligible and all cell biomass could be produced within a day. For the calculation presented here, we used an average CUE of 0.6 based on genome-scale metabolic predictions (Saifuddin et al., 2019). To estimate the number of cells (*N*_*cell*_) that could feed on degraded mucilage, we divided the cell biomass carbon by an average bacterial cell carbon mass (*M*_*cell*_) of 10 fg C per cell (*N_*cell*_* = *M_*B/*_M_*cell*_*) (Khachikyan et al., 2019).

Furthermore, we estimated the EPS produced (*M*_*EPS*_) by a given bacterial abundance by using an EPS yield per unit of cellular biomass (*M_*EPS*_* = *M_*B*_* × *Y*_*EPS*_ with *Y_*EPS*_* = 10 mg g^–1^) (Shene et al., 2008). Our model calculation assumed that all cells produce EPS.

### Statistical Analyses

All data were analyzed by IBM SPSS Statistics for Windows, version 25 (IBM Corp., Armonk, NY, United States). The data were tested for homogeneity of variance and normality by Levene’s test and Shapiro–Wilk test, respectively, and transformed logarithmically if they did not fulfill these prerequisites. The Independent Samples *t*-test was used to test for significant differences between mucilage and EPS in terms of the investigated properties at the significance level of 0.05. All charts were designed using SigmaPlot 14.0 (Systat, San José, CA, United States).

## Results

### Viscosity and Surface Tension

**FIGURE 1:**
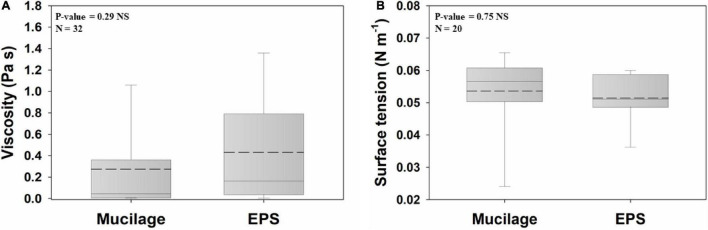
Comparison of mucilage and EPS viscosity **(A)** and surface tension **(B)**, using Independent Samples *t*-test at the significance level of 0.05. The dashed and solid lines on each box indicate the arithmetic mean and median, respectively. The box defines the 25th and 75th percentiles. NS, non-significant; N, number of data points. The viscosity values had been measured at a solute concentration of 0.5 mg ml^–1^, shear rate of 0.5 s^–1^, and temperature of 20–25°C, and the surface tension values had been measured at a solute concentration of 0.5 mg ml^–1^ and temperature of 20–25°C. Viscosity of water at 25°C = 0.00089 Pa s; Surface tension of water at 25°C = 0.072 N m^–1^.

The viscosity of mucilage and EPS did not differ significantly (Figure 1A). Average viscosities for mucilage and EPS were 0.27 Pa s and 0.43 Pa s, respectively. There was also no significant difference between the surface tension of mucilage and EPS (Figure 1B). Average surface tensions for mucilage and EPS were 0.053 N m^–1^ and 0.051 N m^–1^, respectively.

### Total Polysaccharide and Protein

**FIGURE 2:**
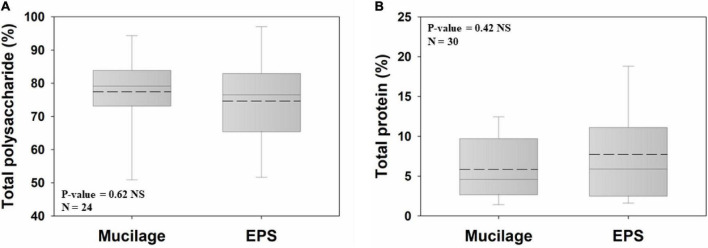
Comparison of mucilage and EPS total polysaccharide **(A)** and total protein **(B)**, using Independent Samples *t*-test at the significance level of 0.05. The dashed and solid lines on each box indicate the arithmetic mean and median, respectively. The box defines the 25th and 75th percentiles. NS, non-significant; N, number of data points.

Polysaccharides were the major chemical constituent of both biogels (77.4% and 74.6% for mucilage and EPS, respectively) and did not significantly differ between them (Figure 2A). The same was true for the total protein proportions of both biogels, being on average 5.8% and 7.7% in mucilage and EPS, respectively (Figure 2B).

### Neutral Monosaccharide and Uronic Acid Composition

**FIGURE 3:**
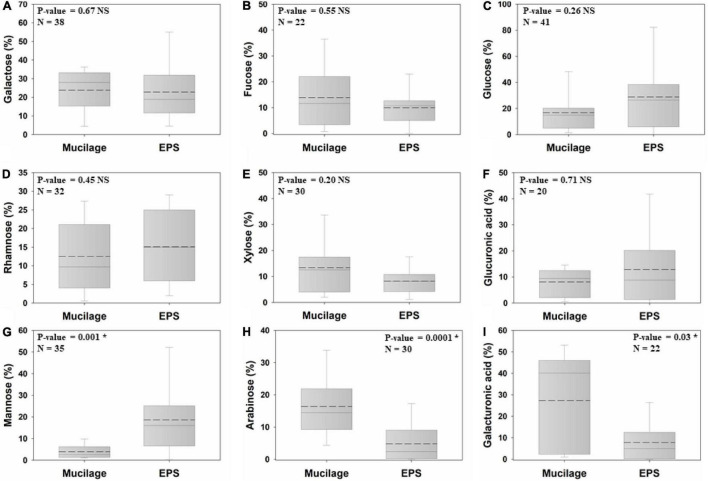
Comparison of the proportion of mucilage and EPS galactose **(A)**, fucose **(B)**, glucose **(C)**, rhamnose **(D)**, xylose **(E)**, glucuronic acid **(F)**, mannose **(G)**, arabinose **(H)**, and galacturonic acid **(I)**, using Independent Samples at the significance level of 0.05. The dashed and solid lines on each box indicate the arithmetic mean and median, respectively. The box defines the 25th and 75th percentiles. NS and * indicate a non-significant and significant difference, respectively. N: number of data points.

Six out of nine studied monomers of the biogels’ polysaccharide backbone did not significantly differ in proportion between mucilage and EPS, namely galactose (mucilage = 23.8%; EPS = 22.8%), fucose (13.9%; 9.9%), glucose (16.7%; 28.7%), rhamnose (12.4%; 15%), xylose (13.4%; 8.1%), and glucuronic acid (8%; 12.8%). In contrast, mannose (3.9%; 18.6%) was significantly higher in EPS than in mucilage (nearly fivefold higher), whereas arabinose (16.3%; 4.8%) and galacturonic acid (27.3%; 7.8%) had higher proportions (3.4-fold and 3.5-fold higher, respectively) in mucilage than in EPS (Figures 3A–I).

### Spatial and Temporal Organization of Mucilage Around Growing Roots

**FIGURE 4:**
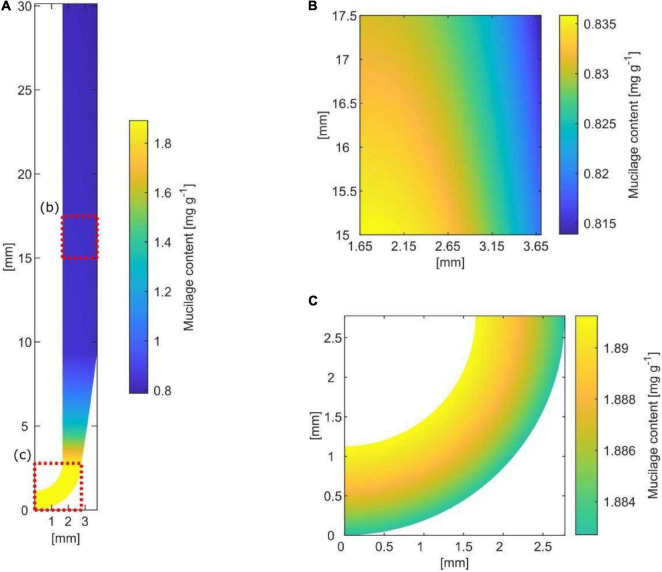
Spatial and temporal distribution of mucilage around a root segment grown within 1 day (= 30 mm) [**(A)** along the root; **(B)** lateral; **(C)** at the root tip]. Mucilage-affected soil is indicated by yellow to blue color along the root and by yellow to green at the root tip, reflecting the increasing radial extent by swelling as well as the decreasing content of mucilage by decomposition.

Based on simplified assumptions of root growth, exudation rate, decomposition rate, hydration ratio, and mucilage expansion into the soil, a simple model for the size and extent of a potential “mucilage biofilm” was developed (Figure 4). The axial rhizosphere extent directly at the root tip was 1.12 mm with a mucilage content of 1.89 mg g^–1^ soil. The kinetic of water saturation and swelling is rapid with an average 6 h until the mucilage of root tips reaches its constant volume. Therefore, 9.2 mm above the root tip, the mucilage is fully hydrated, reaching its final radial extent of 2.05 mm. By swelling, the content decreases to 0.8 mg g^–1^ soil, a value hardly changing by decomposition along the daily grown segment of 30 mm. Assuming a linear decomposition rate of 50% in 7 days, mucilage is only half decayed at a distance 21 cm above the root tip.

### Microbial Abundance and Extracellular Polymeric Substances Production in the Mucilage Matrix

We consider that two different zones around the root receive degradable carbon at varying rates: (i) the zone within 1.65 mm from the root tip where the estimated total mucilage C exudation rate (C_in___rc_) is 5 μg d^–1^, and (ii) the 28.35 mm root zone above the root tip (grown within 1 day) where the mucilage concentration is reduced by gel swelling and degradation (C_inez_ = 122 μg d^–1^). The high C concentrations at the root tip allow for a maximum number of bacterial cells growing on the basis of mucilage C consumption, which is in the order of 10^9^ cells per day. In contrast, lower C concentrations at the 28.35 mm zone above the root tip resulted in only 3 × 10^10^ bacterial cells grown per day. Despite its 25 times larger volume, the 28.35 mm root above the root tip can only host around 10 times more bacterial cells than the small soil volume surrounding the 1.65 mm of the root tip. The bacterial cells could potentially produce around 0.01 μg EPS per day at the root tip and 0.29 μg EPS per day within the 28.35 mm zone of daily growth. Assuming the above given mucilage exudation of 1.41 mg d^–1^ and root tip with a 50% decomposition in 7 days (i.e., 7.1% decomposition per day), 101 μg of mucilage covering the root gets decomposed and replaced by 0.3 μg of EPS per day, assuming mucilage as the sole C source. This leads to a relatively small but continuous modification and thinning of the biofilm along the root axis.

## Discussion

Although individual plant and microbial species and their physiological conditions crucially affect the biogels produced, our study revealed an overall high degree of similarity in the physical and chemical properties of EPS and mucilage. The selected physical and chemical properties control many of the beneficial attributes of EPS, such as the maintenance of hydraulic connectivity, the formation of aggregates or the reduction of enzyme, carbon and nutrient losses. Therefore, the similarity in these physical and chemical properties suggests that mucilage can also function as a biofilm matrix.

Although our study found wide variability among the investigated plant and microbial species, the physical and chemical properties of EPS and mucilage varied only within a moderate range. Hence, both biogels offer similar soil microbial habitats, shaped by vegetation type and soil conditions. In the following, we discuss how the physical and chemical characteristics of mucilage provide three substantial prerequisites for biofilm formation: a moist environment, a protective barrier, and carbon and nutrient provision (Flemming and Wingender, 2010; Velmourougane et al., 2017).

### Biogels as Microbial Habitats

Our results demonstrate that microbial EPS have high viscosity and low surface tension. These two key physical properties of EPS can play important roles in the formation and persistence of microbial biofilms in soils (Lieleg et al., 2011; Benard et al., 2019). High viscosity and low surface tension facilitate adhesion and cohesion of biofilms to mineral or organic surfaces in the soil, bridging microbial cells for biofilm development, and aggregating soil particles (Flemming and Wingender, 2010; Costa et al., 2018). The physical properties of EPS imply several protecting functions against antibiotics, disinfectants, heavy metals, and even against harmful effects of oxygen, by reducing the diffusion of these compounds toward the microbial cells (Flemming and Wingender, 2010). Furthermore, the enhanced soil water retention and liquid-phase connectivity provided by EPS protect microorganisms against drought but also against deep frost (Bore et al., 2017; Benard et al., 2019). Last but not least, viscos EPS can protect against grazing protozoa by adhering to their cilia and blocking their feeding apparatus (Liu and Buskey, 2000; Flemming and Wingender, 2010).

The results of our analyses showed that the viscosity and surface tension of mucilage and EPS are not significantly different. The similarity of mucilage to EPS in terms of these physical characteristics implies that mucilage can provide a biofilm-like habitat to support the life and survival of microorganisms in soils, specifically in the rhizosphere. Numerous studies have confirmed that mucilage provides a moist environment and protective barrier against abiotic and biotic stresses. Mucilage increases the water content of the rhizosphere, connects soil particles, increases the soil liquid-phase connectivity, and facilitates root water uptake under drought, due to its high viscosity and low surface tension (Young, 1995; Carminati et al., 2010; Ahmed et al., 2015; Benard et al., 2019; Zarebanadkouki et al., 2019). Mucilage absorbs 27–589 times more water than its dry weight (McCully and Boyer, 1997; Huang and Gutterman, 1999; Capitani et al., 2013; Nazari et al., 2020), which is considerably higher than the amount of water absorbed by a similar quantity of EPS. The binding of negatively charged uronic acids to Ca^2+^ governs hydration-dehydration dynamics in biogels (Dean et al., 2007; North et al., 2014; Brax et al., 2019). The results of our study indicate rather similar proportions of glucuronic acid in mucilage and EPS but significantly higher galacturonic acid in mucilage. This high proportion of galacturonic acid in mucilage is likely one of the major reasons for the higher water absorption capacity of mucilage than EPS. In addition to hydraulic functions, mucilage is cohesive and adhesive to surfaces and therefore, similar to EPS, improves soil aggregation in the rhizosphere through strengthening bonds between soil particles (Czarnes et al., 2000).

Mucilage ameliorates heavy metals toxicity in the rhizosphere, protects roots against salinity, and functions as a barrier against harmful effects of oxygen (Horst, 1995; Zarebanadkouki et al., 2019). It also traps pathogenic and herbivorous insects and protects microbial symbionts (Haughn and Western, 2012; Galloway et al., 2020). Moreover, an 8 mm layer of crude maize mucilage maintained very low oxygen levels (below 5%) (Van Deynze et al., 2018), which is very similar to oxygen levels in bacterial biofilms (Wessel et al., 2014; Wang et al., 2017). The low oxygen levels in the mucilage can support a microaerobic environment but can also promote crucial functions like nitrogenase activity (Van Deynze et al., 2018; Bennett et al., 2020). It can be deduced that mucilage, like EPS, provides a moist environment and a protective barrier for soil microorganisms in order to form biofilms.

### Association of Chemical Composition and Biogel Functions

Generally, polysaccharides and proteins are major components of EPS and mucilage (Chaboud and Rougier, 1991; Wingender et al., 2001; Flemming and Wingender, 2010; Lembre et al., 2012; Behbahani et al., 2017; Pandit et al., 2020), and our analyses reveal a similar proportional contribution of these components to both biogels. The combination of EPS polysaccharides and proteins is important for the formation, organization, and stability of the biofilm (Flemming and Wingender, 2010; Fong and Yildiz, 2015; Limoli et al., 2015; Shukla and Rao, 2017). Both substance classes likely jointly contribute to the high viscosity of EPS and mucilage and to their low surface tension (Benard et al., 2019). Our analyses also show similar proportions of polysaccharides and proteins, with similar viscosities and surface tensions. This suggests that mucilage can also act in biofilm formation, organization, and stability.

Furthermore, EPS and mucilage have other common properties such as enzymes, extracellular DNA (eDNA), and lipids. However, the proportion of these constituents is low (Nazari, 2021). EPS contain enzymes and eDNA produced by microorganisms inhabiting the biofilm. EPS enzymes can degrade matrix biopolymers such as polysaccharides and proteins in order to provide microorganisms with carbon and energy, a process occurring in EPS mainly under carbon starvation (Costa et al., 2018). This can become a central process of microbial C supply in a plant-provided mucilage biofilm matrix. In the case of the mucilage biofilm, biogel-producing and consuming organisms are different and the “biofilm producer” is an autotrophic organism generally not suffering from low C supply. Like EPS, mucilage was also shown to contain several enzyme classes active in the degradation of major mucilage polysaccharides, releasing monosaccharides such as galactose, mannose, fucose, xylose, and arabinose (Pozzo et al., 2018; Voiniciuc et al., 2018; Bennett et al., 2020), supporting the concept of mucilage as a microbial C source.

Extracellular polymeric substances eDNA increases the structural stability of biofilms, functions as an important agent of microbial aggregation, and acts as an intercellular connector (Molin and Tolker-Nielsen, 2003; Yang et al., 2007; Flemming and Wingender, 2010). Mucilage also comprises eDNA that increases the stability of mucilage and protects root tips against pathogenic infection (Wen et al., 2009; Hawes et al., 2016; Ropitaux et al., 2020).

Lipids play a part in the hydrophobicity of EPS and help microorganisms adhere to waxy, plastic (e.g., Teflon), and pyrite surfaces (Neu and Poralla, 1988; Neu et al., 1992). Similar functions of lipids were described for mucilage, e.g., they control mucilage hydrophobicity and thus the interaction of mucilage with soil solids, water, and transported ions (Read et al., 2003; Chen and Arye, 2017; Nazari, 2021). Mucilage turns hydrophobic upon drying (Ahmed et al., 2016), a process that causes water repellency in the rhizosphere and prevents hydraulic failure in the rhizosphere under drought (Carminati, 2013; Zickenrott et al., 2016). This may be an important mechanism protecting the rhizosphere microbiome from drought effects and maintaining their activity even under water limitation (Ahmed et al., 2018a).

Our study also analyzed the monomer composition of polysaccharides, which are the quantitatively dominant fraction in both biogels. Our results indicated that EPS and mucilage have similar proportions of galactose, fucose, glucose, rhamnose, xylose, and glucuronic acid, while the proportions of mannose, arabinose, and galacturonic acid significantly differed. EPS galactose, fucose, and arabinose play an important role in the enhancement, dispersion, and stability of biofilms (Imberty et al., 2004; Tielker et al., 2005; Diggle et al., 2006; Johansson et al., 2008; Byrd et al., 2009; Ma et al., 2009). Pel and Psl are two major polysaccharides generally present in EPS, which play essential roles in biofilm establishment (Vu et al., 2009; Flemming and Wingender, 2010). Pel is mainly composed of glucose, while Psl is rich in mannose, glucose and rhamnose (Byrd et al., 2009). Thus, the higher mannose content of EPS than mucilage can presumably be explained by the higher proportion of Psl in soil biofilms. Pel and Psl are also necessary for the formation, adherence, and attachment of biofilms to abiotic and biotic surfaces and also for the stability of biofilm architecture (Byrd et al., 2009; Ma et al., 2009; Franklin et al., 2011; Zhurina et al., 2014). Pel and Psl often display high functional redundancy, which suggests that the relative proportions of glucose vs. mannose and rhamnose have rather minor functional implications in EPS (Colvin et al., 2011). This suggests that also variations in these sugars between mucilage and EPS may be of minor functional relevance. In contrast to Psl and Pel, alginate is an anionic polysaccharide of EPS responsible for trapping of cations, a decisive process in biofilm establishment. For instance, calcium functions like a bridge between alginate molecules, leading to thick and compact biofilms with enhanced mechanical stability (Körstgens et al., 2001). The significantly higher proportion of galacturonic acid in mucilage than in EPS suggests that the alginate-related features of EPS are more pronounced and essential in mucilage. Most relevant here is the subsequent increase in extrinsic Ca^2+^ bridges in mucilage, which connect the uronic acids and therefore increase the mucilage stability (Moore and Fondren, 1988; Brax et al., 2019). Thus, this study suggests that the mechanical stability of biogels may differ as a result of the higher galacturonic acid proportion in mucilage than in EPS.

Moreover, uronic acids, being present in the form of their carboxylate anions in soils, may also function as buffering agents in the rhizosphere under extremely acidic conditions. Although carboxylate anions (oxalate, citrate, malate, etc.) released at the zone of maximal root exudation also buffer the proton exudation from the root, these compounds are of low molecular weight and are consequently rapidly decomposed. In contrast, galacturonic acid with a logarithmic acid dissociation constant (pKa) of 3.5 might therefore more efficiently and over a larger zone of the mucilage-covered rhizosphere fulfill this buffering function, at least in very acidic soils. This might be a mechanism contributing to avoiding a very strong rhizosphere acidification and thus a limitation of microbial life and activity (Malik et al., 2018).

In summary, we found a remarkably similar chemical composition of mucilage and EPS, which supports the contention that mucilage can function as a biofilm matrix. Although the individual role of each chemical mucilage component and its impact on microbial life has not yet been unraveled, it is unlikely that the limited deviation in chemical composition between mucilage and EPS would significantly diminish the potential of mucilage to function as a biofilm matrix.

### Mucilage as a Nutrient and Carbon Source for Microorganisms

All EPS components are a potential source of nutrients (Flemming and Wingender, 2010). Since EPS and mucilage share many compositional similarities, mucilage can also be decomposed and consumed by microorganisms. Enzymatic release of highly abundant sugars in mucilage such as galactose, fucose, and arabinose can feed microorganisms residing in the mucilage (Bennett et al., 2020). The presence of endogenous glycosyl hydrolase enzymes in mucilage, which release the terminal fucose and arabinose residues, further augments this claim (Pozzo et al., 2018). Other studies also reported that microorganisms utilize mucilage as an energy source (Mary et al., 1993; Ahmed et al., 2018a,b; Veelen et al., 2018), with average times of 7–15 days for the consumption of 50% of the mucilage carbon added to the soil (Ahmed et al., 2018a). The high protein content of mucilage leads to a C:N ratio of approximately 16:1 (Mary et al., 1993), which is approximately double the C:N ratio of microorganisms (between 7:1 and 8.6:1) (Cleveland and Liptzin, 2007; Xu et al., 2013). Thus, considering that 50% of the C is utilized *via* catabolism and oxidized to gain energy (Manzoni et al., 2012), mucilage has the ideal composition to function as a sole energy, C and N source for microorganisms. Consequently, microorganisms solely need to be supplied with mineral nutrients (P, K, Ca, Mg, etc.)—a common interest shared with their mucilage-providing plants. Our analyses strongly support the claim that mucilage is used as a source of nutrients covering the C and N demand of microorganisms and enabling high growth rates in the mucilage-covered rhizosphere. It is important to note that mucilage can provide a moist environment and protective barrier for EPS-producing but also for non-EPS-producing rhizosphere microorganisms, which can attach to solid surfaces but without formation of biofilms. Similarly, a study in bulk soil revealed that non-EPS-producing microorganisms can also benefit from the biogel produced by EPS-producing microorganisms (Chew and Yang, 2017). However, EPS production can consume large proportions of the available energy of a microbial cell. It can thus be considered as a bioenergetically “expensive” process for microorganisms. Plants, as photoautotrophs, are (partly) in the soil and yet have access to photosynthetically fixed C and thus can invest in extracellular biogels more easily than heterotrophic microorganisms. Mucilage can synergistically support EPS-producing as well as non-EPS-producing microorganisms in the rhizosphere and even overlapping biogel production of microbial EPS and mucilage can occur (Carminati and Vetterlein, 2013). This mucilage-EPS interaction can further boost the formation and modification of biofilms in the rhizosphere with advantageous functions for microorganisms and the plant.

### Spatial and Temporal Implications of Mucilage Matrix for Microbial Life Around the Root

**FIGURE 5:**
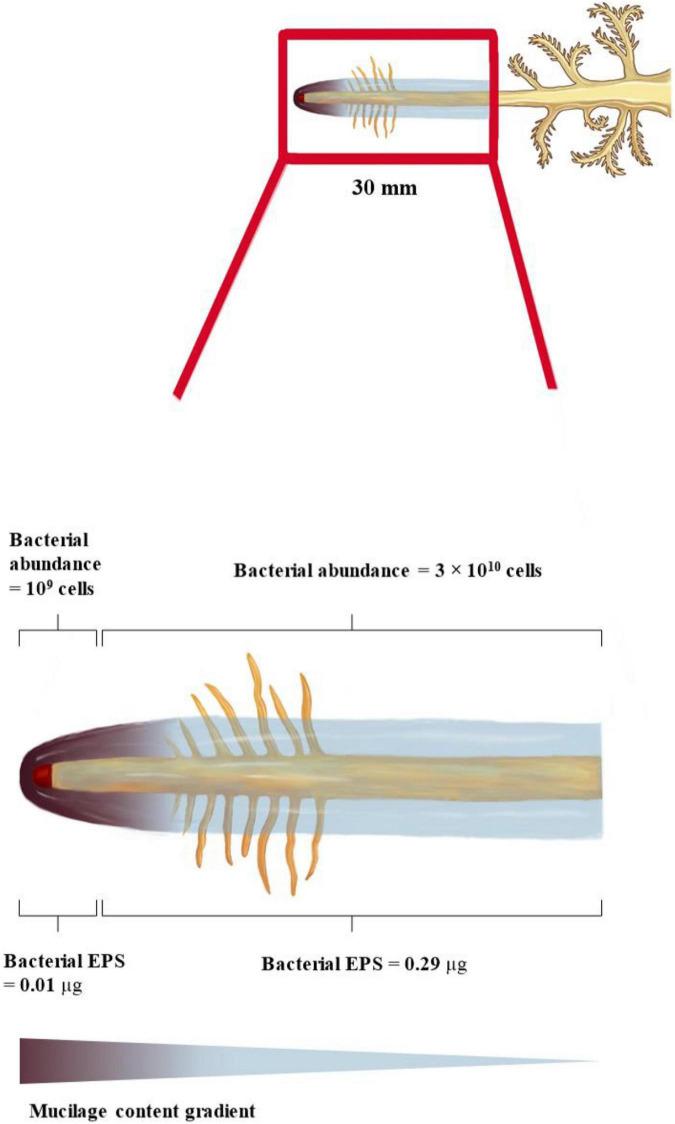
Spatial and temporal model of mucilage biofilm and its bacterial abundance and EPS production along a maize root segment grown within 1 day (= 30 mm). Mucilage content decreases away from the root tip due to bacterial decomposition. Mucilage extent increases away from the root tip due to swelling.

Holz et al. (2018) measured the mucilage distribution of approximately 1 mm around the root. Considering the influence of porosity on the radial extent of mucilage, our estimated maximum mucilage distribution of 2.05 mm around the root is well in agreement with the measured mucilage distribution of Holz et al. (2018). The quantitative relevance of mucilage as a biofilm matrix is defined by the radial and axial extent around the root, and the latter is largely defined by its decomposition kinetics. The maximal decomposition of 50% mucilage within 7 days of incubation under optimal conditions (Ahmed et al., 2018a), assuming a linear decomposition rate, suggests that only 7.14% of the daily mucilage production gets decomposed per day. Therefore, the axial extent of mucilage can reach several decimeters above the root tip without substantial thinning of the mucilage by decomposition (Figure 5).

The contribution of EPS to the root-covering biogel was already suggested by Carminati and Vetterlein (2013), but experimental or analytical studies quantifying the contribution of both biofilm matrices to the rhizosphere biofilm are still lacking. Our estimation of maximal EPS production capacity suggests that only a minor proportion (0.3%) of the decomposed mucilage is replaced by EPS, assuming mucilage as the sole C source (Figure 5). However, the proportion of EPS producers as well as their EPS production rate might be underestimated by our input data derived from pure culture isolates, and excluding fungal EPS. However, especially in the root elongation zone, a few millimeters above the root tip, the exudation of low-molecular-weight substances provides an additional carbon source to be potentially utilized for EPS production (Yang and Crowley, 2000; Sasse et al., 2018; Rüger et al., 2021). Averaging the scarce data available on root exudation rates or amounts suggest low-molecular-weight exudate quantities in the range of 2.4 × 10^–7^ g d^–1^ cm^–1^ (Oburger et al., 2013; Gunina and Yakov, 2015). Even assuming all of this C is readily available for microbial utilization (Sasse et al., 2018; Rüger et al., 2021), this daily release of C is still a magnitude lower than the C provided by the mucilage decomposition (8 × 10^–6^ g C d^–1^ cm^–1^). This suggests that low-molecular-weight exudates may play a minor role as C substrate for EPS production, but more importantly, that their function as a microbial C source might have been overestimated compared to mucilage C.

An approximation of the maximal number of bacterial cells growing on the decomposed mucilage C resulted in 10^10^ cells per cm^3^ mucilage-affected rhizosphere volume, or 10^9^ bacterial cells per g mucilage-affected rhizosphere soil (Figure 5). Although only a limited number of studies have quantified absolute bacterial abundance in the rhizosphere, most of them through gene copy numbers gained by qPCR, 10^9^ is a realistic number for bacterial abundance (Zhu et al., 2016). This suggests that even under the assumption of only moderate decomposition (∼7% per day), mucilage C can function as a major C source supporting a high bacterial abundance in the rhizosphere, potentially without losing its function as a biofilm matrix for several decimeters along the root axis.

The production of EPS requires cellular resources and may be costly for microorganisms (Jayathilake et al., 2017). Hence, their fitness and competitiveness are reduced compared to non-EPS-forming microorganisms if no further environmental stress provides EPS producers with ecological advantage (Vardharajula and Sk, 2014). Therefore, an EPS-based biofilm with the extent of the mucilage-covered rhizosphere volume is impossible for heterotrophic EPS producers in soils. Compared to EPS production by heterotrophic soil microorganisms, mucilage as a biogel does not exhaust soil C sources, but is formed from the photosynthetically fixed C of the autotrophic plant. Considering the bioeconomy of the plant-microbe system, the direct production of the biogel by the autotrophic organism is more efficient than exuding low-molecular-weight C resources for heterotrophic organisms, of which only a minor proportion will be invested in biogel biosynthesis. Consequently, plant mucilage production is an efficient C investment in the context of the whole plant-soil continuum providing (a) a stable habitat for the establishment of the rhizo-microbial community; and (b) a major C source supporting the observed abundances of microbial cells in the rhizosphere zone (Sasse et al., 2018; Rüger et al., 2021). Both properties are “services” provided by the autotrophic plant to boost the rhizosphere microbiome toward high cell densities and highly active microorganisms. Consequently, the microbiological features of the immediate rhizosphere can be linked to mucilage, providing a biofilm matrix for the rhizosphere microbiome. Nonetheless, EPS are likely still important in the rhizosphere, because rhizosphere bacteria capable of producing EPS are associated with better root colonization (Costa et al., 2018; Knights et al., 2021).

## Conclusion

Our analyses revealed similar chemical composition and physical properties in plant mucilage and microbial EPS. This suggests that many functions of mucilage and EPS are comparable and consequently supports the potential of plant mucilage to function as a biofilm matrix similar to EPS. However, in contrast to an EPS-based biofilm, the high rhizosphere C investment required to form the biofilm matrix does not need to be covered by heterotrophic soil microorganisms. Instead, this functional C is provided directly by the autotrophic plant. As autotrophic organisms with substantially higher biomass, the proportional investment of plants in mucilage C is magnitudes lower than for an EPS-producing microbial colony that produce their own biofilm matrix. Therefore, mucilage exudation may be a major contributor to soil biogels, forming large volumes of stable microbial habitats around plant roots. The rhizosphere microbiome is protected against environmental stresses like drought besides being supplied with moderately available C that supports a high rhizosphere microbial abundance. The rather slow decomposition of mucilage compared to the rapid growth of roots leads to axial root segments in the decimeter range surrounded by this unique mucilage-based microhabitat. Therefore, we recommend a reconsideration of mucilage not only as a physical matrix that affects rhizosphere hydraulics, but as a biofilm matrix that supports the rhizosphere microbiome and its resistance to environmental stresses.

## Data Availability Statement

The datasets presented in this study can be found in online repositories. The names of the repository/repositories and accession number(s) can be found in the article/Supplementary Material.

## Author Contributions

MN and MD proposed the idea. MN performed the literature review and comparative analyses, and wrote the manuscript draft. SB developed the bacterial abundance and EPS production model. PB and AC developed the mucilage exudation model. KM-J shared ideas and scientifically enriched the text. MD supervised the project. All authors read the manuscript draft, commented on it, and confirmed it before submission.

## Conflict of Interest

The authors declare that the research was conducted in the absence of any commercial or financial relationships that could be construed as a potential conflict of interest.

## Publisher’s Note

All claims expressed in this article are solely those of the authors and do not necessarily represent those of their affiliated organizations, or those of the publisher, the editors and the reviewers. Any product that may be evaluated in this article, or claim that may be made by its manufacturer, is not guaranteed or endorsed by the publisher.

## Footnotes

1
https://automeris.io/WebPlotDigitizer
