# Supplementary material for: Biogels in Soils: Plant Mucilage as a Biofilm Matrix That Shapes the Rhizosphere Microbial Habitat
Source: Front Plant Sci. 2022 Jan 13;12:798992. doi: 10.3389/fpls.2021.798992 (PMC8792611; doi:10.3389/fpls.2021.798992)
Supplement: Supplementary file 1 [file Data_Sheet_1.docx]

**Table 1.** Investigated physical properties of the biogels produced by different plant and microbial species.

| **Species** | **Biogel source** | **Biogel type** | **Properties** | **References** |
| --- | --- | --- | --- | --- |
| *Zea mays* L.  *Zea mays* L.  *Capsella bursa-pastoris* L.  *Salvia hispanica* L.  *Zea mays* L.  *Hordeum vulgare* L.  *Salvia hispanica* L.  *Linum usitatissimum*  *Salvia hispanica* L.  *Zea mays* L.  *Camelina sativa*  *Linum usitatissimum*  *Salvia hispanica* L.  *Linum usitatissimum*  *Linum usitatissimum*  *Linum usitatissimum*  Zea mays L.  *Lupinus angustifolius* L.  *Sinapis alba* L.  *Sinapis alba* L.  *Salvia hispanica* L.  *Zea mays* L.  *Hordeum vulgare* L.  *Plantago major*  *Triticum aestivum* L.  *Sinapis alba* L.  *Sphingomonas paucimobilis*  *Alcaligenes faecalis*  *Pseudomonas spp.*  *Bacillus spp.*  *Volcaniella eurihalina*  *Bacillus polymyxa*  *Rhizobium tropici*  *Cryptococcus flavus*  *Rhodotorula minuta*  *Bacillus pumilu*  *Anabaena spp.*  *Halomonas eurihalina*  *Oscillatoria formosa*  *Halomonas xianhensis*  *Anabaena circinalis*  *Stephanopyxis turris*  Bacillus spp.  *Chemoorganotrophic B.4*  *Chemoorganotrophic B.18*  *Chemoorganotrophic H.44*  *Chemoorganotrophic J13*  *Chemoorganotrophic J17*  *Pseudomonas fluorescens*  *Planococcus maitriensis*  *Pseudomonas spp.*  Pseudomonas spp. | Root  Root  Seed  Seed  Root  Root  Seed  Seed  Seed  Root  Seed  Seed  Seed  Seed  Seed  Seed  Root  Root  Seed  Seed  Seed  Root  Root  Seed  Root  Seed  Bacterial  Bacterial  Bacterial  Bacterial  Bacterial  Bacterial  Bacterial  Fungal  Fungal  Bacterial  Cyanobacterial  Bacterial  Cyanobacterial  Bacterial  Cyanobacterial  Algal  Bacterial  Bacterial  Bacterial  Bacterial  Bacterial  Bacterial  Bacterial  Bacterial  Bacterial  Bacterial | Mucilage  Mucilage  Mucilage  Mucilage  Mucilage  Mucilage  Mucilage  Mucilage  Mucilage  Mucilage  Mucilage  Mucilage  Mucilage  Mucilage  Mucilage  Mucilage  Mucilage  Mucilage  Mucilage  Mucilage  Mucilage  Mucilage  Mucilage  Mucilage  Mucilage  Mucilage  EPS  EPS  EPS  EPS  EPS  EPS  EPS  EPS  EPS  EPS  EPS  EPS  EPS  EPS  EPS  EPS  EPS  EPS  EPS  EPS  EPS  EPS  EPS  EPS  EPS  EPS | Viscosity  Viscosity  Viscosity  Viscosity  Viscosity  Viscosity  Viscosity  Viscosity  Viscosity  Viscosity  Viscosity  Viscosity  Viscosity  Viscosity  Viscosity  Viscosity  Surface tension  Surface tension  Surface tension  Surface tension  Surface tension  Surface tension  Surface tension  Surface tension  Surface tension  Surface tension  Viscosity  Viscosity  Viscosity  Viscosity  Viscosity  Viscosity  Viscosity  Viscosity  Viscosity  Viscosity  Viscosity  Viscosity  Viscosity  Viscosity  Viscosity  Viscosity  Surface tension  Surface tension  Surface tension  Surface tension  Surface tension  Surface tension  Surface tension  Surface tension  Surface tension  Surface tension | Reed and Gregory 1997  Read et al 1999  Deng et al 2013  Naveed et al 2019  Naveed et al 2019  Naveed et al 2019  Goh et al 2016  Naran et al 2008  Capitani et al 2015  Gregory 2000  Sarv et al 2017  Mazza and Biliaderis 1989  Capitani et al 2016  Kaewmanee et al 2014  Wannerberger et al 1991  Wannerberger et al 1992  Reed and Gregory 1997  Reed and Gregory1997  Weber et al 1974  Wu et al 2015  Naveed et al 2019  Naveed et al 2019  Naveed et al 2019  Alizadeh Behbahani et al 2017  Read et al 2003  Cui et al 2006  Ashtaputre and Shah 1995  Kaur et al 2013  Subramanian et al 2010  Subramanian et al 2010  Quesada et al 1993  Lee et al 1997  Castellane et al 2014  Pavlova et al 2009  Pavlova et al 2009  Chowdhury et al 2011  Moreno et al 2000  Martínez-Checa et al 2002  Jindal et al 2011  Biswas et al 2015  Mancuso Nichols et al 2009  Mancuso Nichols et al 2009  Tansel and Tansel 2013  Warscheid et al 1991  Warscheid et al 1991  Warscheid et al 1991  Warscheid et al 1991  Warscheid et al 1991  Meliani and Bensoltane 2014  Kumar et al 2007  Yongrui et al 2015  Yongrui et al 2015 |

**Table 2.** Investigated chemical compositions of the biogels produced by different plant and microbial species.

| **Species** | **Biogel source** | **Biogel type** | **Composition** | **References** |
| --- | --- | --- | --- | --- |
| *Actinidin chinensis*  *Linum usitatissimum* L.  *Lallemantia royleana*  *Zea mays* L.  *Linum usitatissimum* L.  *Plantago major*  *Eruca sativa*  *Lepidium perfoliatum*  *Lepidium sativum*  *Ocimum basilicum* L.  *Cydonia oblonga*  *Salvia macrosiphon*  *Actinidin chinensis*  *Linum usitatissimum* L.  *Lallemantia royleana*  *Zea mays* L.  *Linum usitatissimum* L.  *Citrus jambheri Lush*  *Sachtion citrumelo*  *Yuma citrange*  *Zea mays* L.  *Plantago major*  *Eruca sativa*  *Lepidium perfoliatum*  *Lepidium sativum*  *Ocimum basilicum* L.  *Cydonia oblonga*  *Salvia macrosiphon*  *Pisum sativum* L.  *Arabidopsis thaliana* L.  *Glossostemon bruguieri*  *Glossostemon bruguieri*  *Kosteletzkya virginica*  *Oryza sativa* L.  *Lepidium sativum*  *Linum usitatissimum* L.  *Actinidin chinensis*  *Linum usitatissimum* L.  *Plantago spp.*  *Zea mays* L.  *Zea mays* L.  *Zea mays* L.  *Linum usitatissimum* L.  *Coffea arabica* L.  *Lallemantia royleana*  *Zea mays L.*  *Zea mays L.*  *Zea mays L.* | Root  Seed  Seed  Root  Seed  Seed  Seed  Seed  Seed  Seed  Seed  Seed  Root  Seed  Seed  Root  Seed  Seed  Seed  Seed  Root  Seed  Seed  Seed  Seed  Seed  Seed  Seed  Root  Seed  Seed  Root  Root  Root  Root  Seed  Root  Seed  Seed  Root  Root  Root  Seed  Seed  Seed  Root  Root  Root | Mucilage  Mucilage  Mucilage  Mucilage  Mucilage  Mucilage  Mucilage  Mucilage  Mucilage  Mucilage  Mucilage  Mucilage  Mucilage  Mucilage  Mucilage  Mucilage  Mucilage  Mucilage  Mucilage  Mucilage  Mucilage  Mucilage  Mucilage  Mucilage  Mucilage  Mucilage  Mucilage  Mucilage  Mucilage  Mucilage  Mucilage  Mucilage  Mucilage  Mucilage  Mucilage  Mucilage  Mucilage  Mucilage  Mucilage  Mucilage  Mucilage  Mucilage  Mucilage  Mucilage  Mucilage  Mucilage  Mucilage  Mucilage | Polysaccharides  Polysaccharides  Polysaccharides  Polysaccharides  Polysaccharides  Polysaccharides  Polysaccharides  Polysaccharides  Polysaccharides  Polysaccharides  Polysaccharides  Polysaccharides  Proteins  Proteins  Proteins  Proteins  Proteins  Proteins  Proteins  Proteins  Proteins  Proteins  Proteins  Proteins  Proteins  Proteins  Proteins  Proteins  Sugars  Sugars  Sugars  Sugars  Sugars  Sugars  Sugars  Sugars  Sugars  Sugars  Sugars  Sugars  Sugars  Sugars  Sugars  Sugars  Sugars  Sugars  Sugars  Sugars | Redgwell 1983  Barbary et al 2009  Alizadeh Behbahani et al 2018  Chaboud and Rougier 1991  Paynel et al 2013  Alizadeh Behbahani et al 2017  Koocheki et al 2012  Koocheki et al 2013  Karazhiyan et al 2011  Razavi et al 2009  Jouki et al 2014  Farahnaky et al 2013  Redgwell 1983  Barbary et al 2009  Behbahani et al 2018  Chaboud and Rougier 1991  Uschapovsky et al 2015  Naqvi et al 2011  Naqvi et al 2011  Naqvi et al 2011  Gould and Northcote 1986  Alizadeh Behbahani et al 2017  Koocheki et al 2012  Koocheki et al 2013  Karazhiyan et al 2011  Razavi et al 2009  Jouki et al 2014  Farahnaky et al 2013  Knee et al 2001  Rautengarten et al 2008  Ibrahim et al 1997  Ibrahim et al 1997  Ghanem et al 2010  Chaboud and Rougier 1984  Ray et al 1998  Fedeniuk and Biliaderis 1994  Redgwell 1983  Barbary et al 2009  Phan et al 2016  Amicucci et al 2019  Van Deynze et al 2018  Osborn et al 1999  Wannerberger et al 1991  Avallone et al 2000  Alizadeh Behbahani et al 2018  Chaboud 1983  Bacic et al 1986  Gould and Northcote 1986 |

| **Species** | **Biogel source** | **Biogel type** | **Composition** | **References** |
| --- | --- | --- | --- | --- |
| *Pseudomonas fluorescens*  *Pseudomonas fluorescens*  *Klebsiella pneumoniae*  *Rahnella aquatilis*  *Brevundimonas vesicularis*  *Geitlerinema spp.*  *Plectonema cf. battersii*  *Chroococcus submarinus*  *Rhabdoderma cf. rubrum*  *Breundimonas esicularis sp.*  *Cryptococcus flavus*  *Bacillus licheniformis*  *Pseudomonas fluorescens*  *Pseudomonas fluorescens*  *Sagittula stellata*  *Sagittula stellata*  *Geitlerinema spp.*  *Plectonema cf. battersii*  *Chroococcus submarinus*  *Rhabdoderma cf. rubrum*  *Breundimonas esicularis sp.*  *Microcystis spp.*  *Volcaniella eurihalina*  *Pseudomonas fluorescens*  *Cryptococcus flavus*  *Pseudoalteromonas spp.*  *Bacillus licheniformis*  *Schizothrix spp.*  *Acidophilic Spp.*  *Klebsiella pneumoniae*  *Rahnella aquatilis*  *Brevundimonas vesicularis*  *Synechocystis sp.*  *Oscillatoria sp.*  *Breundimonas vesicularis sp.*  *Volcaniella eurihalina*  *Synechocystis sp.*  *Synechocystis sp.*  *methanogenic granules*  *Methanobacterium formicicum*  *Methanobacterium formicicum*  *Bacillus coagulans*  *Thalassiosira sp.*  *Lactobacillus pentosus*  *Lactobacillus casei*  *Pseudomonas aeruginosa*  *Pseudomonas aeruginosa*  *Pseudomonas stutzeri*  *Achnanthes longipes*  *Amphora coffeaeformis*  *Cymbella cistula*  *Burkholderia cepacia*  *Burkholderia gladioli*  *Pseudomonas fluorescens*  *Cryptococcus flavus*  *Pseudoalteromonas spp.* | Bacterial  Bacterial  Bacterial  Bacterial  Bacterial  Cyanobacterial  Cyanobacterial  Cyanobacterial  Cyanobacterial  Bacterial  Fungal  Bacterial  Bacterial  Bacterial  Bacterial  Bacterial  Cyanobacterial  Cyanobacterial  Cyanobacterial  Cyanobacterial  Bacterial  Cyanobacterial  Bacterial  Bacterial  Fungal  Bacterial  Bacterial  Cyanobacterial  Bacterial  Bacterial  Bacterial  Bacterial  Cyanobacterial  Cyanobacterial  Bacterial  Bacterial  Cyanobacterial  Cyanobacterial  Bacterial  Bacterial  Bacterial  Bacterial  Algal  Bacterial  Bacterial  Bacterial  Bacterial  Bacterial  Algal  Algal  Algal  Bacterial  Bacterial  Bacterial  Fungal  Bacterial | EPS  EPS  EPS  EPS  EPS  EPS  EPS  EPS  EPS  EPS  EPS  EPS  EPS  EPS  EPS  EPS  EPS  EPS  EPS  EPS  EPS  EPS  EPS  EPS  EPS  EPS  EPS  EPS  EPS  EPS  EPS  EPS  EPS  EPS  EPS  EPS  EPS  EPS  EPS  EPS  EPS  EPS  EPS  EPS  EPS  EPS  EPS  EPS  EPS  EPS  EPS  EPS  EPS  EPS  EPS  EPS | Polysaccharides  Polysaccharides  Polysaccharides  Polysaccharides  Polysaccharides  Polysaccharides  Polysaccharides  Polysaccharides  Polysaccharides  Polysaccharides  Polysaccharides  Polysaccharides  Proteins  Proteins  Proteins  Proteins  Proteins  Proteins  Proteins  Proteins  Proteins  Proteins  Proteins  Proteins  Proteins  Proteins  Proteins  Proteins  Sugars  Sugars  Sugars  Sugars  Sugars  Sugars  Sugars  Sugars  Sugars  Sugars  Sugars  Sugars  Sugars  Sugars  Sugars  Sugars  Sugars  Sugars  Sugars  Sugars  Sugars  Sugars  Sugars  Sugars  Sugars  Sugars  Sugars  Sugars | Xu et al 2011  Xu et al 2011  Verhoef et al 2005  Verhoef et al 2005  Verhoef et al 2005  Richert et al 2005  Richert et al 2005  Richert et al 2005  Richert et al 2005  Verhoef et al 2002  Pavlova et al 2009  Spano et al 2013  Xu et al 2011  Xu et al 2011  Xu et al 2011  Xu et al 2011  Richert et al 2005  Richert et al 2005  Richert et al 2005  Richert et al 2005  Verhoef et al 2002  Li et al 2009  Bejar et al 1996  Ceyhan and Ozdemir 2008  Pavlova et al 2009  Nichols et al 2005  Spano et al 2013  Kawaguchi and Decho 2002  Jiao et al 2010  Verhoef et al 2005  Verhoef et al 2005  Verhoef et al 2005  Kawaguchi and Decho 2000  Kawaguchi and Decho 2000  Verhoef et al 2002  Bejar et al 1996  Ozturk and Aslim 2010  Ozturk and Aslim 2010  Veiga et al 1997  Veiga et al 1997  Veiga et al 1997  Kodali et al 2000  Giroldo et al 2003  Sanchez et al 2006  Kojic et al 1992  Onbasli and Aslim 2009  Onbasli and Aslim 2009  Onbasli and Aslim 2009  Wustman et al 1997  Wustman et al 1997  Wustman et al 1997  Ceyhan and Ozdemir 2008  Ceyhan and Ozdemir 2008  Ceyhan and Ozdemir 2008  Pavlova et al 2009  Nichols et al 2005 |

**Table 2.** Continued.

**References**

Alizadeh Behbahani, B. A., & Imani Fooladi, A. A. (2018). Shirazi balangu (Lallemantia royleana) seed mucilage: Chemical composition, molecular weight, biological activity and its evaluation as edible coating on beefs. *International Journal of Biological Macromolecules*. https://doi.org/10.1016/j.ijbiomac.2018.03.177

Alizadeh Behbahani, B., Tabatabaei Yazdi, F., Shahidi, F., Hesarinejad, M. A., Mortazavi, S. A., & Mohebbi, M. (2017). Plantago major seed mucilage: Optimization of extraction and some physicochemical and rheological aspects. *Carbohydrate Polymers*. https://doi.org/10.1016/j.carbpol.2016.08.051

Amicucci, M. J., Galermo, A. G., Guerrero, A., Treves, G., Nandita, E., Kailemia, M. J., Higdon, S. M., Pozzo, T., Labavitch, J. M., Bennett, A. B., & Lebrilla, C. B. (2019). Strategy for Structural Elucidation of Polysaccharides: Elucidation of a Maize Mucilage that Harbors Diazotrophic Bacteria. *Analytical Chemistry*. https://doi.org/10.1021/acs.analchem.9b00789

Ashtaputre, A. A., & Shah, A. K. (1995). Studies on a viscous, gel-forming exopolysaccharide from Sphingomonas paucimobilis GS1. *Applied and Environmental Microbiology*. https://doi.org/10.1128/aem.61.3.1159-1162.1995

Avallone, S., Guiraud, J. P., Guyot, B., Olguin, E., & Brillouet, J. M. (2000). Polysaccharide constituents of coffee-bean mucilage. *Journal of Food Science*. https://doi.org/10.1111/j.1365-2621.2000.tb10602.x

Bacic, A., Moody, S. F., & Clarke, A. E. (1986). Structural Analysis of Secreted Root Slime from Maize ( Zea mays L.) . *Plant Physiology*. https://doi.org/10.1104/pp.80.3.771

Bala Subramanian, S., Yan, S., Tyagi, R. D., & Surampalli, R. Y. (2010). Extracellular polymeric substances (EPS) producing bacterial strains of municipal wastewater sludge: Isolation, molecular identification, EPS characterization and performance for sludge settling and dewatering. *Water Research*. https://doi.org/10.1016/j.watres.2009.12.046

Barbary, O.M., Al-Sohaimy, S.A., El-Saadani, M.A., 2009. Extraction, composition and physicochemical properties of flaxseed mucilage. J. Adv. Agric. Res. 14 (3), 605

Bejar, V., Calvo, C., Moliz, J., Diaz-Martinez, F., & Quesada, E. (1996). Effect of growth conditions on the rheological properties and chemical composition of Volcaniella eurihalina exopolysaccharide. *Applied Biochemistry and Biotechnology - Part A Enzyme Engineering and Biotechnology*. https://doi.org/10.1007/BF02787859

Biswas, J., Mandal, S., & Paul, A. K. (2015). Production, Partial Purification and Some Bio-physicochemical Properties of EPS Produced by Halomonas xianhensis SUR308 Isolated from a Saltern Environment. *Journal of Biologically Active Products from Nature*. https://doi.org/10.1080/22311866.2015.1038852

Capitani, M. I., Corzo-Rios, L. J., Chel-Guerrero, L. A., Betancur-Ancona, D. A., Nolasco, S. M., & Tomás, M. C. (2015). Rheological properties of aqueous dispersions of chia (Salvia hispanica L.) mucilage. *Journal of Food Engineering*. https://doi.org/10.1016/j.jfoodeng.2014.09.043

Capitani, M. I., Nolasco, S. M., & Tomás, M. C. (2016). Stability of oil-in-water (O/W) emulsions with chia (Salvia hispanica L.) mucilage. *Food Hydrocolloids*. https://doi.org/10.1016/j.foodhyd.2016.06.008

Castellane, T. C. L., Lemos, M. V. F., & Lemos, E. G. D. M. (2014). Evaluation of the biotechnological potential of Rhizobium tropici strains for exopolysaccharide production. *Carbohydrate Polymers*. https://doi.org/10.1016/j.carbpol.2014.04.066

Ceyhan, N., & Ozdemir, G. (2008). Extracellular polysaccharides produced by cooling water tower biofilm bacteria and their possible degradation. *Biofouling*. https://doi.org/10.1080/08927010801911316

Chaboud, A. (1983). Isolation, purification and chemical composition of maize root cap slime. *Plant and Soil*. https://doi.org/10.1007/BF02184316

Chaboud, A., & Rougier, M. (1991). Effect of Root Density in Incubation Medium on Root Exudate Composition of Axenic Maize Seedlings. *Journal of Plant Physiology*. https://doi.org/10.1016/S0176-1617(11)80706-6

Chowdhury, S. R., Basak, R. K., Sen, R., & Adhikari, B. (2011). Characterization and emulsifying property of a carbohydrate polymer produced by Bacillus pumilus UW-02 isolated from waste water irrigated agricultural soil. *International Journal of Biological Macromolecules*. https://doi.org/10.1016/j.ijbiomac.2011.02.019

Cui, S. W., Eskin, M. A. N., Wu, Y., & Ding, S. (2006). Synergisms between yellow mustard mucilage and galactomannans and applications in food products - A mini review. In *Advances in Colloid and Interface Science*. https://doi.org/10.1016/j.cis.2006.11.012

Deng, W., Iannetta, P. P. M., Hallett, P. D., Toorop, P. E., Squire, G. R., & Jeng, D. S. (2013). The rheological properties of the seed coat mucilage of Capsella bursa-pastoris L. Medik. (shepherd’s purse). *Biorheology*. https://doi.org/10.3233/BIR-130627

Edmond Ghanem, M., Han, R. M., Classen, B., Quetin-Leclerq, J., Mahy, G., Ruan, C. J., Qin, P., Pérez-Alfocea, F., & Lutts, S. (2010). Mucilage and polysaccharides in the halophyte plant species Kosteletzkya virginica: Localization and composition in relation to salt stress. *Journal of Plant Physiology*. https://doi.org/10.1016/j.jplph.2009.10.012

Farahnaky, A., Shanesazzadeh, E., Mesbahi, G., & Majzoobi, M. (2013). Effect of various salts and pH condition on rheological properties of Salvia macrosiphon hydrocolloid solutions. *Journal of Food Engineering*. https://doi.org/10.1016/j.jfoodeng.2013.01.036

Fedeniuk, R. W., & Biliaderis, C. G. (1994). Composition and Physicochemical Properties of Linseed (Linum usitatissimum L.) Mucilage. *Journal of Agricultural and Food Chemistry*. https://doi.org/10.1021/jf00038a003

Giroldo, D., Henriques Vieira, A. A., & Paulsen, B. S. (2003). relative increase of deoxy sugars during microbial degradation of an extracellular polysaccharide released by a tropical freshwater Thalassiosira sp. (Bacillariophyceae). *Journal of Phycology*. https://doi.org/10.1111/j.0022-3646.2003.03-006.x

Goh, K. K. T., Matia-Merino, L., Chiang, J. H., Quek, R., Soh, S. J. B., & Lentle, R. G. (2016). The physico-chemical properties of chia seed polysaccharide and its microgel dispersion rheology. *Carbohydrate Polymers*. https://doi.org/10.1016/j.carbpol.2016.04.126

Gould, J., & Northcote, D. H. (1986). Cell-cell recognition of host surfaces by pathogens. The adsorption of maize (Zea mays) root mucilage by surfaces of pathogenic fungi. *The Biochemical Journal*. https://doi.org/10.1042/bj2330395

Gregory, P. J. (2000). Physical changes in the rhizosphere and their significance for plant-soil interactions. In *Acta Agronomica Hungarica*. https://doi.org/10.1556/AAgr.48.2000.1.12

Ibrahim, N., El-Eraky, W., El-Gengaihi, S., & Shalaby, A. S. (1997). Chemical and biological evaluation of proteins and mucilages from roots and seeds of Glossostemon bruguieri Desf. (Moghat). *Plant Foods for Human Nutrition*. https://doi.org/10.1007/BF02436043

Jiao, Y., Cody, G. D., Harding, A. K., Wilmes, P., Schrenk, M., Wheeler, K. E., Banfield, J. F., & Thelen, M. P. (2010). Characterization of extracellular polymeric substances from acidophilic microbial biofilms. *Applied and Environmental Microbiology*. https://doi.org/10.1128/AEM.02289-09

Jindal, N., Singh, D. P., & Khattar, J. I. S. (2011). Kinetics and physico-chemical characterization of exopolysaccharides produced by the cyanobacterium Oscillatoria formosa. *World Journal of Microbiology and Biotechnology*. https://doi.org/10.1007/s11274-011-0678-6

Jouki, M., Mortazavi, S. A., Yazdi, F. T., & Koocheki, A. (2014). Optimization of extraction, antioxidant activity and functional properties of quince seed mucilage by RSM. *International Journal of Biological Macromolecules*. https://doi.org/10.1016/j.ijbiomac.2014.02.026

Kaewmanee, T., Bagnasco, L., Benjakul, S., Lanteri, S., Morelli, C. F., Speranza, G., & Cosulich, M. E. (2014). Characterisation of mucilages extracted from seven Italian cultivars of flax. *Food Chemistry*. https://doi.org/10.1016/j.foodchem.2013.10.022

Karazhiyan, H., Razavi, S. M. A., Phillips, G. O., Fang, Y., Al-Assaf, S., & Nishinari, K. (2011). Physicochemical aspects of hydrocolloid extract from the seeds of Lepidium sativum. *International Journal of Food Science and Technology*. https://doi.org/10.1111/j.1365-2621.2011.02583.x

Kaur, V., Bera, M., Panesar, P., & Chopra, H. (2013). Production and Characterization of Exopolysaccharide Produced by Alcaligenes Faecalis B14 Isolated from Indigenous Soil. *International Journal of Biotechnology and Bioengineering Research*.

Kawaguchi, T., & Decho, A. W. (2000). Biochemical characterization of cyanobacterial Extracellular Polymers (EPS) from modern marine stromatolites (Bahamas). *Preparative Biochemistry and Biotechnology*. https://doi.org/10.1080/10826060008544971

Kawaguchi, T., & Decho, A. W. (2002). A laboratory investigation of cyanobacterial extracellular polymeric secretions (EPS) in influencing CaCO3 polymorphism. *Journal of Crystal Growth*. https://doi.org/10.1016/S0022-0248(02)00918-1

Knee, E. M., Gong, F. C., Gao, M., Teplitski, M., Jones, A. R., Foxworthy, A., Mort, A. J., & Bauer, W. D. (2001). Root mucilage from pea and its utilization by rhizosphere bacteria as a sole carbon source. *Molecular Plant-Microbe Interactions*. https://doi.org/10.1094/MPMI.2001.14.6.775

Kodali, V. P., Das, S., & Sen, R. (2009). An exopolysaccharide from a probiotic: Biosynthesis dynamics, composition and emulsifying activity. *Food Research International*. https://doi.org/10.1016/j.foodres.2009.02.007

Kojic, M., Vujcic, M., Banina, A., Cocconcelli, P., Cerning, J., & Topisirovic, L. (1992). Analysis of exopolysaccharide production by Lactobacillus casei CG11, isolated from cheese. *Applied and Environmental Microbiology*. https://doi.org/10.1128/aem.58.12.4086-4088.1992

Koocheki, A., Razavi, S. M. A., & Hesarinejad, M. A. (2012). Effect of Extraction Procedures on Functional Properties of Eruca sativa Seed Mucilage. *Food Biophysics*. https://doi.org/10.1007/s11483-011-9245-9

Koocheki, A., Taherian, A. R., & Bostan, A. (2013). Studies on the steady shear flow behavior and functional properties of Lepidium perfoliatum seed gum. *Food Research International*. https://doi.org/10.1016/j.foodres.2011.05.002

Kumar, A., Mody, K., & Jha, B. (2007). Evaluation of biosurfactant/bioemulsifier production by a marine bacterium. *Bulletin of Environmental Contamination and Toxicology*. https://doi.org/10.1007/s00128-007-9283-7

Lee, I. Y., Seo, W. T., Kim, G. J., Kim, M. K., Ahn, S. G., Kwon, G. S., & Park, Y. H. (1997). Optimization of fermentation conditions for production of exopolysaccharide by Bacillus polymyxa. *Bioprocess Engineering*. https://doi.org/10.1007/s004490050290

Li, P., Cai, Y., Shi, L., Geng, L., Xing, P., Yu, Y., Kong, F., & Wang, Y. (2009). Microbial degradation and preliminary chemical characterization of Microcystis Exopolysaccharides from a Cyanobacterial water bloom of Lake Taihu. *International Review of Hydrobiology*. https://doi.org/10.1002/iroh.200911149

Mancuso Nichols, C. A., Nairn, K. M., Glattauer, V., Blackburn, S. I., Ramshaw, J. A. M., & Graham, L. D. (2009). Screening microalgal cultures in search of microbial exopolysaccharides with potential as adhesives. *Journal of Adhesion*. https://doi.org/10.1080/00218460902782071

Martínez-Checa, F., Toledo, F., Vilchez, R., Quesada, E., & Calvo, C. (2002). Yield production, chemical composition, and functional properties of emulsifier H28 synthesized by Halomonas eurihalinastrain H-28 in media containing various hydrocarbons. *Applied Microbiology and Biotechnology*. https://doi.org/10.1007/s00253-001-0903-6

[Mazza](https://onlinelibrary.wiley.com/action/doSearch?ContribAuthorStored=MAZZA%2C+G), G. &  [Biliaderis](https://onlinelibrary.wiley.com/action/doSearch?ContribAuthorStored=BILIADERIS%2C+C+G), G. (1989). Functional properties of flax seed mucilage. *Journal of Food Science.* <https://doi.org/10.1111/j.1365-2621.1989.tb05978.x>

Meliani, A. (2014). Enhancement of Hydrocarbons Degradation by Use of Pseudomonas Biosurfactants and Biofilms. *Journal of Petroleum & Environmental Biotechnology*. https://doi.org/10.4172/2157-7463.1000168

Moreno, J., Vargas, M. A., Madiedo, J. M., Muñoz, J., Rivas, J., & Guerrero, M. G. (2000). Chemical and rheological properties of an extracellular polysaccharide produced by the cyanobacterium Anabaena sp. ATCC 33047. *Biotechnology and Bioengineering*. https://doi.org/10.1002/(SICI)1097-0290(20000205)67:3<283::AID-BIT4>3.0.CO;2-H

Naqvi, S. A., Khan, M. M., Shahid, M., Jaskani, M. J., Khan, I. A., Zuber, M., & Zia, K. M. (2011). Biochemical profiling of mucilage extracted from seeds of different citrus rootstocks. *Carbohydrate Polymers*. https://doi.org/10.1016/j.carbpol.2010.08.031

Naran, R., Chen, G., & Carpita, N. C. (2008). Novel rhamnogalacturonan I and arabinoxylan polysaccharides of flax seed mucilage. *Plant Physiology*. https://doi.org/10.1104/pp.108.123513

Naveed, M., Ahmed, M. A., Benard, P., Brown, L. K., George, T. S., Bengough, A. G., Roose, T., Koebernick, N., & Hallett, P. D. (2019). Surface tension, rheology and hydrophobicity of rhizodeposits and seed mucilage influence soil water retention and hysteresis. *Plant and Soil*. https://doi.org/10.1007/s11104-019-03939-9

Nichols, C. M., Lardière, S. G., Bowman, J. P., Nichols, P. D., Gibson, J. A. E., & Guézennec, J. (2005). Chemical characterization of exopolysaccharides from Antarctic marine bacteria. *Microbial Ecology*. https://doi.org/10.1007/s00248-004-0093-8

Onbasli, D., & Aslim, B. (2009). Effects of some organic pollutants on the exopolysaccharides (EPSs) produced by some Pseudomonas spp. strains. *Journal of Hazardous Materials*. https://doi.org/10.1016/j.jhazmat.2009.01.131

Osborn, H. M. I., Lochey, F., Mosley, L., & Read, D. (1999). Analysis of polysaccharides and monosaccharides in the root mucilage of maize (Zea mays L.) by gas chromatography. *Journal of Chromatography A*. https://doi.org/10.1016/S0021-9673(98)00935-2

Ozturk, S., & Aslim, B. (2010). Modification of exopolysaccharide composition and production by three cyanobacterial isolates under salt stress. *Environmental Science and Pollution Research*. https://doi.org/10.1007/s11356-009-0233-2

Pavlova, K., Panchev, I., Krachanova, M., & Gocheva, M. (2009). Production of an exopolysaccharide by Antarctic yeast. *Folia Microbiologica*. https://doi.org/10.1007/s12223-009-0049-y

Paynel, F., Pavlov, A., Ancelin, G., Rihouey, C., Picton, L., Lebrun, L., & Morvan, C. (2013). Polysaccharide hydrolases are released with mucilages after water hydration of flax seeds. *Plant Physiology and Biochemistry*. https://doi.org/10.1016/j.plaphy.2012.10.009

Phan, J. L., Tucker, M. R., Khor, S. F., Shirley, N., Lahnstein, J., Beahan, C., Bacic, A., & Burton, R. A. (2016). Differences in glycosyltransferase family 61 accompany variation in seed coat mucilage composition in Plantago spp. *Journal of Experimental Botany*. https://doi.org/10.1093/jxb/erw424

Quesada, E., Bejar, V., & Calvo, C. (1993). Exopolysaccharide production by Volcaniella eurihalina. In *Experientia*. https://doi.org/10.1007/BF01929910

Rautengarten, C., Usadel, B., Neumetzler, L., Hartmann, J., Büssis, D., & Altmann, T. (2008). A subtilisin-like serine protease essential for mucilage release from Arabidopsis seed coats. *Plant Journal*. https://doi.org/10.1111/j.1365-313X.2008.03437.x

Ray, T. C., Callow, J. A., & Kennedy, J. F. (1988). Composition of root mucilage polysaccharides from Lepidium sativum. *Journal of Experimental Botany*. https://doi.org/10.1093/jxb/39.9.1249

Razavi, S. M. A., Mortazavi, S. A., Matia-Merino, L., Hosseini-Parvar, S. H., Motamedzadegan, A., & Khanipour, E. (2009). Optimisation study of gum extraction from Basil seeds (Ocimum basilicum L.). *International Journal of Food Science and Technology*. https://doi.org/10.1111/j.1365-2621.2009.01993.x

Read, D. B., & Gregory, P. J. (1997). Surface tension and viscosity of axenic maize and lupin root mucilages. *New Phytologist*. https://doi.org/10.1046/j.1469-8137.1997.00859.x

Read, D. B., Bengough, A. G., Gregory, P. J., Crawford, J. W., Robinson, D., Scrimgeour, C. M., Young, I. M., Zhang, K., & Zhang, X. (2003). Plant roots release phospholipid surfactants that modify the physical and chemical properties of soil. *New Phytologist*. https://doi.org/10.1046/j.1469-8137.2003.00665.x

Read, D. B., Gregory, P. J., & Bell, A. E. (1999). Physical properties of axenic maize root mucilage. *Plant and Soil*. https://doi.org/10.1023/A:1004403812307

Redgwell, R. J. (1983). Composition of Actinidia mucilage. *Phytochemistry*. https://doi.org/10.1016/0031-9422(83)85028-6

Richert, L., Golubic, S., Le Guédès, R., Ratiskol, J., Payri, C., & Guezennec, J. (2005). Characterization of exopolysaccharides produced by cyanobacteria isolated from Polynesian microbial mats. *Current Microbiology*. <https://doi.org/10.1007/s00284-005-0069-z>

Sánchez, J. I., Martínez, B., Guillén, R., Jiménez-Díaz, R., & Rodríguez, A. (2006). Culture conditions determine the balance between two different exopolysaccharides produced by Lactobacillus pentosus LPS26. *Applied and Environmental Microbiology*. https://doi.org/10.1128/AEM.01078-06

Sarv, V., Trass, O., & Diosady, L. L. (2017). Preparation and Characterization of Camelina sativa Protein Isolates and Mucilage. *JAOCS, Journal of the American Oil Chemists’ Society*. <https://doi.org/10.1007/s11746-017-3031-x>

Spanò, A., Gugliandolo, C., Lentini, V., Maugeri, T. L., Anzelmo, G., Poli, A., & Nicolaus, B. (2013). A novel EPS-producing strain of bacillus licheniformis isolated from a shallow vent Off Panarea Island (Italy). *Current Microbiology*. https://doi.org/10.1007/s00284-013-0327-4

Tansel, B., & Tansel, D. Z. (2013). Adhesion strength and spreading characteristics of EPS on membrane surfaces during lateral and central growth. *Colloids and Surfaces B: Biointerfaces*. https://doi.org/10.1016/j.colsurfb.2013.07.005

Uschapovsky, I. V., Ozhimkova, E. V., Sulman, E. M., Martirosova, E. I., & Plashchina, I. G. (2015). Genetic diversity of flax crop (Linum usitatissimum L.) on glycan-protein composition of seedcoat’s mucilage. *Russian Agricultural Sciences*. https://doi.org/10.3103/s1068367415050237

Van Deynze, A., Zamora, P., Delaux, P. M., Heitmann, C., Jayaraman, D., Rajasekar, S., Graham, D., Maeda, J., Gibson, D., Schwartz, K. D., Berry, A. M., Bhatnagar, S., Jospin, G., Darling, A., Jeannotte, R., Lopez, J., Weimer, B. C., Eisen, J. A., Shapiro, H. Y., … Bennett, A. B. (2018). Nitrogen fixation in a landrace of maize is supported by a mucilage-associated diazotrophic microbiota. *PLoS Biology*. https://doi.org/10.1371/journal.pbio.2006352

Veiga, M. C., Jain, M. K., Wu, W. M., Hollingsworth, R. I., & Zeikus, J. G. (1997). Composition and role of extracellular polymers in methanogenic granules. *Applied and Environmental Microbiology*. https://doi.org/10.1128/aem.63.2.403-407.1997

Verhoef, R., De Waard, P., Schols, H. A., Rättö, M., Siika-Aho, M., & Voragen, A. G. J. (2002). Structural elucidation of the EPS of slime producing Brevundimonas vesicularis sp. isolated from a paper machine. *Carbohydrate Research*. https://doi.org/10.1016/S0008-6215(02)00280-X

Verhoef, R., Schols, H. A., Blanco, A., Siika-Aho, M., Rättö, M., Buchert, J., Lenon, G., & Voragen, A. G. J. (2005). Sugar composition and FT-IR analysis of exopolysaccharides produced by microbial isolates from paper mill slime deposits. *Biotechnology and Bioengineering*. https://doi.org/10.1002/bit.20494

Wannerberger, K., & Nyman, M. (1991). Rheological and Chemical Properties of Mucilage in Different Varieties from Linseed (Linum Usitatissimum). *Acta Agriculturae Scandinavica*. https://doi.org/10.1080/00015129109439914

Warscheid, T., Oelting, M., & Krumbein, W. E. (1991). Physico-chemical aspects of biodeterioration processes on rocks with special regard to organic pollutants. *International Biodeterioration*. https://doi.org/10.1016/0265-3036(91)90032-M

Weber, F. E., Taillie, S. A., & Stauffer, K. R. (1974). Functional characteristics of mustard mucilage. *Journal of Food Science*. https://doi.org/10.1111/j.1365-2621.1974.tb02925.x

Wu, Y., Eskin, N. A. M., Cui, W., & Pokharel, B. (2015). Emulsifying properties of water soluble yellow mustard mucilage: A comparative study with gum Arabic and citrus pectin. *Food Hydrocolloids*. https://doi.org/10.1016/j.foodhyd.2015.01.020

Wustman, B. A., Gretz, M. R., & Hoagland, K. D. (1997). Extracellular matrix assembly in diatoms (Bacillariophyceae): I. A model of adhesives based on chemical characterization and localization of polysaccharides from the marine diatom Achnanthes longipes and other diatoms. *Plant Physiology*. https://doi.org/10.1104/pp.113.4.1059

Xu, C., Zhang, S., Chuang, C. ying, Miller, E. J., Schwehr, K. A., & Santschi, P. H. (2011). Chemical composition and relative hydrophobicity of microbial exopolymeric substances (EPS) isolated by anion exchange chromatography and their actinide-binding affinities. *Marine Chemistry*. https://doi.org/10.1016/j.marchem.2011.03.004

Yongrui., Bao, M., Li, Y., Li, G., Lu, J., & Sun, P. (2015). Characterization of crude oil degrading microbial cultures isolated in Qingdao China. *RSC Advances*. <https://doi.org/10.1039/c5ra16628d>
