# Supplementary material for: Biogels in Soils: Plant Mucilage as a Biofilm Matrix That Shapes the Rhizosphere Microbial Habitat
Source: Front Plant Sci. 2022 Jan 13;12:798992. doi: 10.3389/fpls.2021.798992 (PMC8792611; doi:10.3389/fpls.2021.798992)
Supplement: Supplementary file 2 [file Data_Sheet_2.docx]

**Estimation of mucilage extent and content along a root segment**

$\mathrm{Exudation} rate=1.41 {mg day}^{-1}$

*Exudation rate is the dry weight of mucilage exuded from the root tip per day.*

Growth rate = 30 mm day^-1^

*The increase in root length per day.*

$$Degradation of mucilage to 0.5 of its initial mass:168 hours (7 days)$$

*Fraction of mucilage degraded by microbial activity in one week.*$Root diameter=3.3 mm$

$$Soil porosity=0.5$$

$$Soil mineral density=2.65 \frac{\mathrm{mg}}{\mathrm{mm}^{3}}$$

*Density of the soil solid phase used to derive mucilage content from liquid mucilage concentration and porosity.*

$$Density of hydrated mucilage=1 \frac{\mathrm{mm}^{3}}{\mathrm{mg}}$$

*Since the ratio of dry to wet weight of mucilage is on the order of mg per g we neglect the impact of mucilage concentration on liquid density.*

$$Initial ratio of wet to dry weight of exuded mucilage =\frac{166}{1}$$

*Initial ratio of wet to dry weight of mucilage exuded from the root tip.*

$Initial volume of mucilage exuded in one day=1.41 mg* \frac{166}{1}*1 \frac{\mathrm{mm}^{3}}{\mathrm{mg}}=234 \mathrm{mm}^{3}$

$$Ratio of wet to dry weight of fully hydrated mucilage =\frac{425}{1}$$

*Final ratio of wet to dry weight of mucilage after full hydration i.e., maximum volumetric extent.*

$$Time until full hydration after exudation=6 hours$$

*We assume full hydration of exuded mucilage in 6 hours.*

The following calculations and approximations were done using MATLAB (R2020a). The geometry of a root was assumed as a cylinder as root axis and a semi-spherical cap as root tip. Exudation is assumed at the root tip into the volume of a spherical cap of soil at a constant rate. Mucilage hydration and degradation are derived from an initially uniform distribution of a given volume of mucilage exuded in one day i.e., with the same extent from any point of the root surface.

For the uniform distribution of mucilage and given input parameters, the time to fill the semi-spherical soil volume at the root tip is 100 minutes hence the average age of mucilage in this volume is 50 minutes. Since we assume a linear hydration i.e., swelling of mucilage over time and a constant exudation rate, the hydrated volume of mucilage is derived in this region for its average residence time of 50 minutes. In this time, mucilage swells from its initial wet weight to dry weight ratio of 166/1 to 203/1 (i.e., 16.5 to 20.1 mm^3^). The gradient in mucilage content from the semi-spherical root tip is derived for given degradation rate as a function of distance (which is a function of time) from the root surface.

In the second step, this profile of partly hydrated mucilage extent and mucilage content at the root tip are extended along the root axis. First, mucilage hydration and extent are derived for the average residence time of mucilage along the root axis which is function of growth rate. Then, the gradient in mucilage content from the root surface is calculated for this updated radial extent. Finally, the mucilage content is updated for mucilage age as function of distance from the root surface.

**Reference**

MATLAB. 2020. The MathWorks, Inc. Version 9.8.0.1396136 (R2020a) Update 3
